# Otomycosis: a retrospective study

**DOI:** 10.1016/S1808-8694(15)30653-4

**Published:** 2015-10-19

**Authors:** Zélia Braz Vieira da Silva Pontes, Anna Débora Ferreira Silva, Edeltrudes de Oliveira Lima, Márcio de Holanda Guerra, Neuza Maria Cavalcanti Oliveira, Maria de Fátima Farias Peixoto Carvalho, Felipe Sarmento Queiroga Guerra

**Affiliations:** 1 PhD, Professor; 2 Pharmacist, Biochemist; 3 PhD, Professor; 4 Specialist, MD; 5 MSc., Biochemist, Pharmacist; 6 Specialist, Biochemist, Pharmacist; 7 Pharmacist, Clinical analysis student

**Keywords:** aspergillus, candida, otitis externa

## Abstract

Otomycosis is a fungal infection of the external ear canal with only a few studies about its real frequence in Brazil.

**Aim:**

to evaluate otomycosis frequence and characteristics in patients with clinical suspicion of external otitis.

**Study design:**

Retrospective study with transversal cohort (2000-2006).

**Materials and methods:**

103 patients were assigned to mycological diagnosis (direct microscopic examination and culture).

**Results:**

Otomycosis was diagnosed in 19.4% of the patients. Patient age varied from 2 to 66 years (an average of 23.5 years of age), and 60% of otomycosis cases were seen in women between 2 to 20 years of age. Chronic otitis, previous antibiotic therapy and the lack of cerumen were predisposing factors; itching, otalgia, otorrhea and hypoacusis were the symptoms reported by the patients. The most frequently isolated species were C. albicans (30%), C. parapsilosis (20%), A. niger (20%), A. flavus (10%), A. fumigatus (5%), C. tropicalis (5%), Trichosporon asahii (5%) and Scedosporium apiospermum (5%).

**Conclusions:**

Otomycosis is endemic in JoÆo Pessoa-PB. Clinical exam and mycological studies are important for diagnostic purposes because otomycosis symptoms are not specific.

## INTRODUCTION

It is estimated that otitis externa make up 5 to 20% of ear-related visits to ENTs, most of them caused by bacteria, and from the latter 9 to 25% are caused by fungi, called fungal otitis or otomycosis^1^,^2^. It is an infection that involves the external ear canal squamous epithelium, characterized by pruritus and occasional otalgia and hypoacusis^3^,^4^.

Predisposing factors such as a failure in the ear's defense mechanisms (changes in the coating epithelium, changes in pH, quantitative and qualitative changes in ear wax), bacterial infection, hearing aid or a hearing prosthesis, self-inflicted trauma (use of q-tips to clean the ear), swimming, broad spectrum antibiotic agents, steroids and cytostatic medication, neoplasia and immune disorders, all of which can render the host susceptible to the development of otomycosis^2^,^5^,^6^.

After clinical exam (otoscopy and biomicroscopy) it is possible to confirm diagnosis through mycological exams. Species from genera Aspergillus and Candida are the ones most often involved. These fungi are opportunistic and usually bear varied pathogenicity, being part of the normal microbiota from different body parts^7^,^8^.

Treatment recommendations go from germ termination or controlling predisposing factors, to local debridement (microaspiration) and/or the use of antimicrobial agents (topic/systemic)^9^,^10^.

Although otomycosis is a disease spread throughout the world, there are only a handful of studies regarding its true frequency in Brazil^4^,^11^, ^12^, ^13^, especially in João Pessoa-PB. The present paper aims at assessing otomycosis symptoms and frequency in patients referred to the Mycology Lab for mycological diagnosis.

## MATERIALS AND METHODS

A retrospective, descriptive study, with quantitative analysis was carried out from January of 2000 to December of 2006, based on the records from the Department of Pharmaceutics Mycology Lab, and was approved by the ethics committee (0019/08) of the Health Sciences Center Bioethics Committee of a Federal Higher Education Institution. During this period, 103 patients with clinical suspicion of external otitis coming from the ENT outpatient ward of a University Hospital in the city of João Pessoa - PB were seen.

The biological material collection procedure was innocuous, bringing the patients no risk. Considering that the inner and middle ears are sterile, the external ear bears a skin commensal microbiota, before material collection we cleaned the external ear canal with a moist swab. In case there was secretion in the canal, we used a sterile swab for the collection and the skin scales were collected with the help of a sterile loop.

The samples were processed through a direct microscopic exam (KOH 20% + Quink Parker 51 permanent (2:1)14 and culture in agar Sabouraud dextrose with chloramphenicol (0.05 mg/mL). The cultures were cultivated at 25-37°C with weekly observation during 30 days.

The morphological characteristics of yeasts were identified according to Lodder's criteria (1971)^15^ by the production of germinative tube, hydrolysis and urea, pseudofilaments and clamidoconides, and carbohydrate assimilation and fermentation. The isolates with characteristics of filamentous fungi were identified based criteria from Hoog and Guarro (1999)^16^ by microcultivation.

## RESULTS

A total of 103 patients were referred to mycological diagnosis of otomycosis, with an average of 12.7 requests per year. In 19.4% of the patients the clinical diagnosis of otomycosis was confirmed by direct microscopic examination and repetitive positive cultures.

The age of the 20 patients varied from 2 to 66 years (mean age: 23.5 years) and 60% of them were between 2 and 20 years, and were females (Table I).

**Table 1:** Otomycosis etiological agents according to gender, age and ear affected of 20 patients with clinical suspicion of otitis externa

Fungal species	Otomycosis n (%)	Gender F M	Age	Affected ear Left ear
C. albicans*	6 (30)	5 1	2, 4, 7, 15, 19, 59	4 4
C. parapsilosis*	4 (20)	3 1	2, 14, 15, 45	3 2
C. tropicalis	1 (5)	– 1	33	1 -
T. asahii	1 (5)	1 -	13	– 1
A. niger*	4 (20)	3 1	22, 23, 43, 66	4 1
A. flavus	2 (10)	– 2	18, 51	1 1
A. fumigatus	1 (5)	– 1	13	1 -
S. apiospermum	1 (5)	– 1	5	– 1
TOTAL n (%)	20 (100)	12 (60) 8 (40)		14 (58) 10 (42)

* Bilateral otomycosis case

We observed 40% of bilateral infections and 57% infections in the right ear. Chronic otitis (30%), prior antibiotic treatment (30%), no cerumen (20%) external auditory canal manipulation (15%) were the most relevant predisposing factors; and the most reported clinical signs were: pruritus (60%), otalgia (45%), otorhrea (30%) and hypacusis (30%) (multiple responses).

As germs responsible for these otomycosis cases, Candida genus was the most frequent (55%), followed by Aspergillus (35%), Trichosporon (5%) and Scedosporium (5%). Of the species identified, 30% were C. albicans, 20% C. parapsilosis, 20% A. niger, 10% A. flavus, 5% A. fumigatus, 5% C. tropicalis, 5% T. asahii and 5% S. apiospermum (Table I).

## DISCUSSION

Otomycoses are frequent infections in tropical countries, because of humidity and heat^2^,^17^, ^18^, ^19^. In São Paulo - SP, Brazil of 736 cases of otitis, 2.7% were otomycosis^20^. Notwithstanding, there are very few otomycosis studies in Brazil^4^,^11^, ^12^, ^13^. In João Pessoa - PB, Brazil, of 103 patients with clinical suspicion of otitis externa, 19.4% were diagnosed with otomycosis.

Usually, otomycosis can be diagnosed by means of a clinical exam; nonetheless, a high rate of assumption is required, and the most frequent symptom is pruritus; and otalgia in the most advanced stages, otorrhea and/or hypocusis^7^,^10^,^21^. However, in this study, the diagnose was based on symptoms and laboratory workup; and pruritus, otalgia, otorrhea and/or hypacusis were the symptoms more frequently reported by the patients. These symptoms can be attributed to factors such as humidity and heat recorded in João Pessoa, as well as lack of cerumen by washing the external auditory canal and/or its manipulation reported by the patients, without losing sight of the fact that most of the patients were of low socioeconomical status.

The occurrence of bilateral otomycosis is very low^4^,^7^,^17^. Ho et al. (2006)^10^ observed a bilateral involvement in 7% of the patients, while in this study this figure reached 20%.

The women (60%) in the present study were more often affected by otomycosis, and such figures were closer to those observed by Zaror et al. (1991)^4^ (65%). However, these data are in disagreement from the findings by Kaur et al., (2000)^7^, Ho et al. (2006)^10^ and Yenia et al. (1990)^17^ who found 60%, 56% and 52.5%, respectively in males. Otomycosis was seen in patients aged between 2 and 66 years. Nonetheless, 50% of the cases were diagnosed in patients between 2 and 15 years of age. Occurrences of 70% to 41.1% were seen in patients within the age range of 16 to 30 years^4^,^7^,^17^.

Species of Aspergillus and Candida are the most commonly identified germs causing otomycosis. Studies found a greater prevalence of Aspergillus (A. niger, A. fumigatus, A. flavus and/or Aspergillus spp.) as otomycosis agents^7^,^17^,^18^,^22^, ^23^, ^24^, ^25^. Jaiswal et al. (1990)^26^ and Navarrete et al. (2000)21 found 46% and 35% of Candida spp., respectively. In São Paulo, there were 75% of Aspergillus and 20% of Candida4 species identified. The data found in the present study were of 55% of isolates of Candida (C. albicans, C. parapsilosis and C. tropicalis) and 35% of Aspergillus (A. niger, A. flavus and A. fumigatus).

T. asahii and S. apiospermum were also identified as causing agents in these cases of otomycosis. Reiersöl (1955)^27^ reported a case of otomycosis by T. cutaneum. The Scedosporium genus encompasses a group of filamentous fungi isolated from water, soil, stalled or polluted water all over the world. Two species cause human infection: S. apiospermum (asexual anamorphous of Pseudoallescheria boydii) and S. prolificans (S. inflatum). Considered infrequent, more important as human pathogens, especially in immunocompromised patients^28^,^29^.

A five-year review in Northern England included 3 patients with otitis who had polymicrobial culture, including P. boydii^28^. Yao and Messer (2001)30 diagnosed malignant otitis externa caused by Scedosporium apiospermum in AIDS patients. In immunocompetent patients the fungi affects the tissues, bones or joints after trauma. Otitis media and externa by S. apiospermum was diagnosed in an immunocompetent woman (62 years of age) who had symptoms of chronic otomastoiditis and otorhea^32^. In the present study, S. apiospermum was found in the left external auditory meatus of a five-year old immunocompetent boy.

## CONCLUSION

Otomycosis is, effectively, an endemic disease of João Pessoa-PB, a tropical climate city. Clinical follow up and mycological diagnosis are important since symptoms (pruritus, otalgia, otorrhea and hypacusis) are not specific.
